# Associations between modes of cannabis use and cannabis use disorder: Evidence from the 2022 to 2023 United States National Survey on Drug Use and Health

**DOI:** 10.1111/add.70474

**Published:** 2026-05-26

**Authors:** Amrit Baral, Cerina Dubois, Lakshmi Kumar, Janardan Devkota, Denise C. Vidot, Ryan Vandrey, Johannes Thrul

**Affiliations:** 1 Bloomberg School of Public Health, Johns Hopkins University, Baltimore, MD, USA; 2 Department of Psychiatry and Behavioral Sciences, Johns Hopkins University School of Medicine, Baltimore, MD, USA; 3 Global Cannabis and Psychedelics Research Collaboratory, School of Nursing and Health Studies, University of Miami, Coral Gables, FL, USA; 4 Department of Public Health Sciences, University of Miami Miller School of Medicine, Miami, FL, USA; 5 Sidney Kimmel Comprehensive Cancer Center, Johns Hopkins University, Baltimore, MD, USA; 6 Centre for Alcohol Policy Research, La Trobe University, Melbourne, Australia

**Keywords:** cannabis, cannabis use disorder, marijuana, multi-modal use, NSDUH, routes of administration

## Abstract

**Background and Aims:**

With expanding cannabis legalization, normalization, and diversifying products and delivery methods in the United States (US), cannabis use disorder (CUD) prevalence is rising. Various modes of cannabis use may influence pharmacokinetics, usage patterns, and harm, affecting CUD risk. We measured associations between modes of cannabis use, including multi-modal patterns, and CUD prevalence and severity.

**Design and Setting:**

This cross-sectional study analyzed data from a nationally representative sample of US adults using the 2022–2023 National Survey on Drug Use and Health (NSDUH) data. Multivariable logistic regression analyses were employed to estimate the association between modes of cannabis use and past-year CUD, adjusting for potential confounders and covariates. Analyses were stratified by sex, age, and cannabis use frequency. Among multi-modal users, common combinations and their associations with CUD were further examined.

**Participants/Cases:**

Respondents 18 years or older who reported past-year cannabis use (unweighted n = 25 549; weighted N = 58 850 309).

**Measurements:**

Exposure of interest was the mode of cannabis use, primarily categorized as smoke-only, vape-only, oral/mucosal-only, dab-only, topicals-only, and multi-modal (≥ two modes). The outcome variable was CUD in the past year, and CUD severity, based on *Diagnostic and Statistical Manual of Mental Disorders, Fifth Edition (DSM-5)* criteria. Covariates included age, sex, race/ethnicity, income, education, state cannabis law status, age of cannabis initiation, cannabis use motive, frequency of use, perceived risk of smoking cannabis, illicit drug use, past year mental illness, nicotine dependence, and alcohol use disorder.

**Findings:**

Of the total past-year cannabis users, 53.9% reported multi-modal cannabis use. Overall, CUD prevalence was 30.3%, ranging from 4.4% among oral/mucosal-only to 40.5% among multi-modal, and 28.9% among dab-only users (p < 0.0001). Moderate-to-severe CUD affected 13.2% of all users and was concentrated among multi-modal and dab-only users. In multivariable regression, multi-modal users had fourfold higher odds of CUD (adjusted odds ratio [AOR] = 4.14; 95% confidence interval [CI]: 2.91–5.90). Elevated odds were also observed among smoke-only (AOR = 2.98; 95% CI: 2.02–4.39) and vape/dab-only users (AOR = 1.89; 95% CI: 1.09–3.29), compared with oral/mucosal-only users. Analyses of multi-modal combinations showed the highest CUD odds among those using smoke + vape + oral/mucosal + dab (AOR = 19.74; 95% CI: 9.11–42.75), compared with oral/mucosal + topicals users.

**Conclusions:**

In the United States, modes of cannabis use appear to be statistically significantly associated with prevalence and severity of cannabis use disorder, with multi-modal and inhaled routes conferring the greatest risk. Findings underscore the importance of considering mode of use alongside frequency and potency in clinical assessment, prevention, and policy strategies aimed at reducing cannabis-related harms.

## INTRODUCTION

Cannabis use disorder (CUD) is a mental health condition characterized by a problematic pattern of cannabis use that leads to negative health consequences, including significant impairment or distress [1]. With approximately 54% of the United States (US) adults living in states where cannabis is legal for adult use [2, 3], concerns about CUD have grown. Estimates of CUD prevalence vary by population studied, ranging from approximately 10% among cannabis users globally [1] to 21% among primary care patients reporting past 30-day cannabis use in the US [4]. This increase parallels the rapid expansion of the cannabis marketplace and the diversification of consumption methods [5] that include inhalation (smoking, vaping and dabbing of concentrates), oral ingestion (edibles and beverages), mucosal absorption and topical application [6]. Understanding the routes of administration and combinations of methods is critical because they influence the pharmacokinetics (i.e. absorption, distribution and metabolism) of cannabinoids [7], patterns of use and perceived harm [8], which may shape CUD risk.

Controlled research shows that variation in the route of cannabis administration influences the onset, duration and magnitude of pharmacodynamic effects [9, 10], while reinforcement theory suggests that faster onset and higher potency drug effects can increase rewarding effects and addiction risk [11]. Guided by these frameworks, we posit that multi-modal use, particularly patterns that include inhalation of high-potency Δ-9-tetrahydrocannabinol (THC) concentrates, may confer higher CUD risk and greater CUD severity by combining rapid-onset (vaping and dabbing concentrates or smoking) with prolonged effects from ingested products (e.g. edibles), increasing cumulative THC exposure and reinforcement. In parallel, the growing availability of high-potency inhaled THC products such as concentrates (e.g. wax, shatter and rosin) frequently exceed 70% THC, underscoring potency-related concerns such as tolerance, withdrawal and CUD risk [12], although large surveys typically do not measure potency directly.

Frequency of cannabis use has been extensively studied and is consistently identified as a dominant correlate of CUD risk [13], with markedly higher risk among daily/near-daily users [1]. Despite the growing diversity of cannabis products in the United States, nationally representative evidence on how different modes of cannabis use relate to CUD prevalence and severity remains limited. Prior research by Choi *et al.* [8] using National Survey on Drug Use and Health (NSDUH) data, characterized patterns of cannabis consumption and risk perceptions, but primarily examined perceived risk of smoking cannabis rather than directly estimating CUD risk across modes. Although that study identified associations between perceived smoking risk, use of edibles or drinks and correlations between consumption methods and CUD, it did not directly quantify mode-specific and/or multi-modal CUD risk [8].

Beyond NSDUH, large-scale epidemiologic data linking routes of cannabis use to CUD remain scarce, partly because of the lack of validated tools that capture the evolving complexity of cannabis formulations and delivery systems [14, 15]. Prior studies suggest that route of cannabis use and multi-modal use may relate to CUD risk [16–18], but route-specific evidence remains limited in population-based analysis. Much of the CUD literature instead focuses on treatment modalities, including psychosocial and pharmacological interventions [19] and risk perceptions [20].

To address this gap, the present study places CUD as the primary outcome, systematically examining both its prevalence and severity across individual modes of use and multi-modal combinations. Using the most recent (2022–2023) NSDUH data, we applied a granular classification that distinguishes not only common modes (smoking, oral/mucosal), but also high-potency routes, such as vaping and ‘dabbing’ high-potency concentrates, hypothesized to represent higher-risk consumption profiles.

## METHODS

### Data source and study population

This study used de-identified, publicly available data from the NSDUH 2022 to 2023. Administered annually by the Substance Abuse and Mental Health Services Administration (SAMHSA), the NSDUH uses multi-stage area probability sampling and provides nationally representative cross-sectional estimates of substance use and behavioral health outcomes among the civilian, non-institutionalized US population 12 years and older [21]. Computer-assisted interviewing and audio computer-assisted self-interviewing techniques were used for data collection. Detailed information on survey design and methodology can be found elsewhere [21, 22].

Data used in this study were de-identified by SAMHSA, qualifying the research for exemption from institutional review board (IRB) review. The analytic sample included adults aged 18 years and older who reported past-year cannabis use (unweighted *n* = 25 578, weighted *n* = 58 850 309) (Figure S1). Public-use NSDUH data files are available for download at the SAMHSA website: https://www.samhsa.gov/data/.

### Exposure of interest

The primary exposure was the mode of cannabis use. Respondents were first asked whether they had ever used marijuana or any cannabis product [excluding cannabidiol (CBD) or hemp products]. Those answering ‘yes’ were queried about past-year cannabis use, age at first use and time since last use.

Past-year cannabis use was defined as any cannabis use during the preceding 12 months. Among past-year users, respondents indicated in which ways they used cannabis during the past 12 months, with separate yes/no items for: smoking (e.g. joints, pipes, bongs, blunts and hookahs), vaping (e.g. vape pens, dab pens, tabletop or portable vaporizers), dabbing waxes/shatter/concentrates, eating or drinking, drops/strips/lozenges/sprays in the mouth, applying lotion/cream/patch to skin (topicals), pills and ‘some other way.’ Each mode was coded as a binary variable (1 = yes, 0 = no). We combined eating or drinking, drops/strips/lozenges/sprays in the mouth and pills into a single ‘oral/mucosal’ category. Therefore, single-mode categories were limited to smoking-only, vaping-only, oral/mucosal-only, dabbing-only and topicals-only. The multi-mode group was defined as the use of two or more modes in the past 12 months. Together, this resulted in a six-category cannabis use variable of (1) smoking-only; (2) vaping-only; (3) oral/mucosal-only; (4) dab-only; (5) topicals-only; and (6) multi-modal. We excluded others-only (*n* = 29) from the analyses.

For subgroup analysis within the multi-modal group, we analyzed the most common unique combinations (*n* = 12) based on their distribution: (1) ‘smoke + oral/mucosal’, (2) ‘smoke + vape + oral/mucosal’, (3) ‘smoke + vape + oral/mucosal + dab’, (4) ‘smoke + vape’, (5) ‘smoke + vape + dab’, (6) ‘vape + oral/mucosal’, (7) ‘smoke + dab’, (8) ‘smoke + oral/mucosal + dab’, (9) ‘smoke + vape + oral/mucosal + dab + topical’, (10) ‘smoke + oral/mucosal + topical’, (11) ‘oral/mucosal + topical’, and (12) ‘other combinations’ (see details in Table S1).

### Outcomes of interest

The primary outcomes of interest were past-year CUD and CUD severity, based on Diagnostic and Statistical Manual of Mental Disorders (DSM)-5 criteria [23]. The DSM-5 defines CUD as a ‘problematic pattern of cannabis use leading to clinically significant impairment or distress’ in at least two of 11 domains of functioning within 12 months: tolerance; withdrawal; unsuccessful efforts to cut down or control use; failure to fulfill obligations at work, school or home; continued use despite recurrent physical or psychological consequences of use; and continued cannabis use despite having recurrent social or interpersonal problems caused or exacerbated by the effects of cannabis [24]. In NSDUH, each DSM-5 CUD criterion is assessed using dichotomous (yes/no) domains indicating whether the symptom/behavior occurred in the past 12 months. CUD severity count-based is consistent with DSM-5 thresholds. CUD severity was categorized as mild (endorsement of 2–3 domains), moderate (4–5 domains) or severe (≥6 domains).

### Covariates

Key covariates included: age, sex, race/ethnicity, family income, education level, health insurance, residence status (rurality) and state medical cannabis law status at the time of interview. Cannabis-related variables comprised age at initiation (<18 versus ≥18), indicators of medical use (any medical use, all use medical) and a cannabis-use motive (recreation-only, medical-only or both); days of cannabis use in the past year [near-daily/daily (300–365 days) and non-near-daily/daily (<300 days)] [25]; perceived risk of self-harm, physically and in other ways, by smoking cannabis 1 to 2 times per week; illicit drug use (excluding cannabis); past-month nicotine dependence; past-year alcohol use disorder; any mental illness and any psychotherapeutics (i.e. pain relievers, tranquilizers, stimulants and sedatives) use disorder. See Table S2 for details on the operationalization of variables.

### Statistical analyses

We described the socio-demographic, cannabis use, behavioral and clinical characteristics by cannabis use modes (smoke-only, vape-only, oral/mucosal-only, dab-only, topicals-only and multi-modal), comparing distributions using χ^2^ tests with Rao Scott’s second-order correction and reporting survey-weighted prevalence (Tables 1 and 2).

**FIGURE 1:**
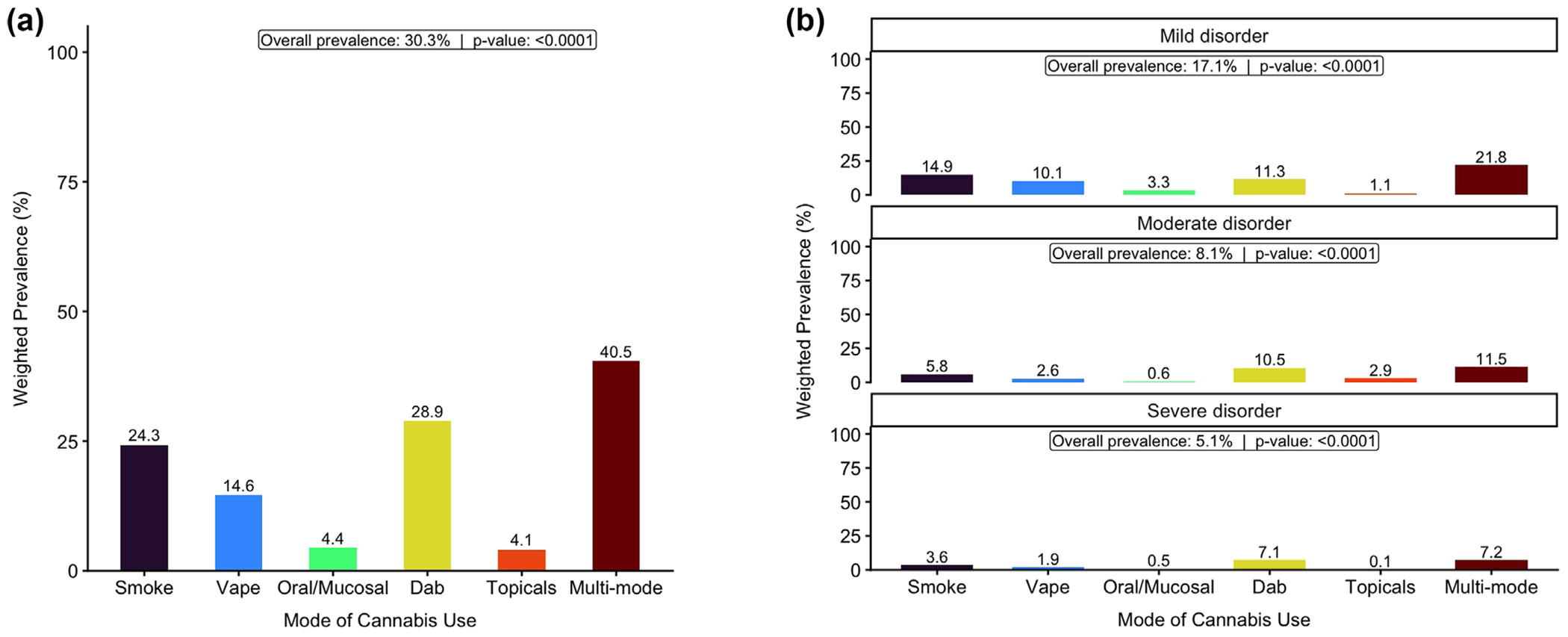
(a),(b) Cannabis use disorder prevalence and severity by method of cannabis use among past-year cannabis users, 18 years and older (unweighted *n* = 25 549, weighted *n* = 58 850 309); 2022–2023 National Survey on Drug Use and Health (NSDUH). Multi-mode cannabis consumption means use of ≥2 methods. Other-only modes (n = 29) were excluded from the analysis. *P-values are calculated using χ^2^ tests with Rao-Scott second-order correction.

Next, we assessed the association of past-year CUD and CUD severity with cannabis use modes using a χ^2^ test with Rao Scott’s second-order correction [Figure 1(a,b), respectively].

Survey-weighted univariable and multi-variable logistic regression estimated the association between modes of cannabis use and past-year CUD in the overall sample, with ‘oral/mucosal-only’ as the reference (Table 3). For regression analyses, vape-only and dab-only users were combined into a single vape/dab-only category to improve estimate stability, given small subgroup sizes in dab-only users, as well as conceptual similarity and shared pharmacologic profile as high-potency products administered via inhalation [26, 27]. Topical-only users were excluded because of limited sample size. Both unadjusted and adjusted odds ratios (ORs) and 95% CIs were reported. Covariates included in the model were age, sex, race/ethnicity, family income, education, health insurance, residence, state medical cannabis law, cannabis initiation age, cannabis-use motive, days cannabis used in the past year, perceived risk of smoking cannabis 1–2 times/week, past-month nicotine dependence and past-year alcohol use disorder, mental illness, illicit drug use and any psychotherapeutics use disorder.

**FIGURE 2:**
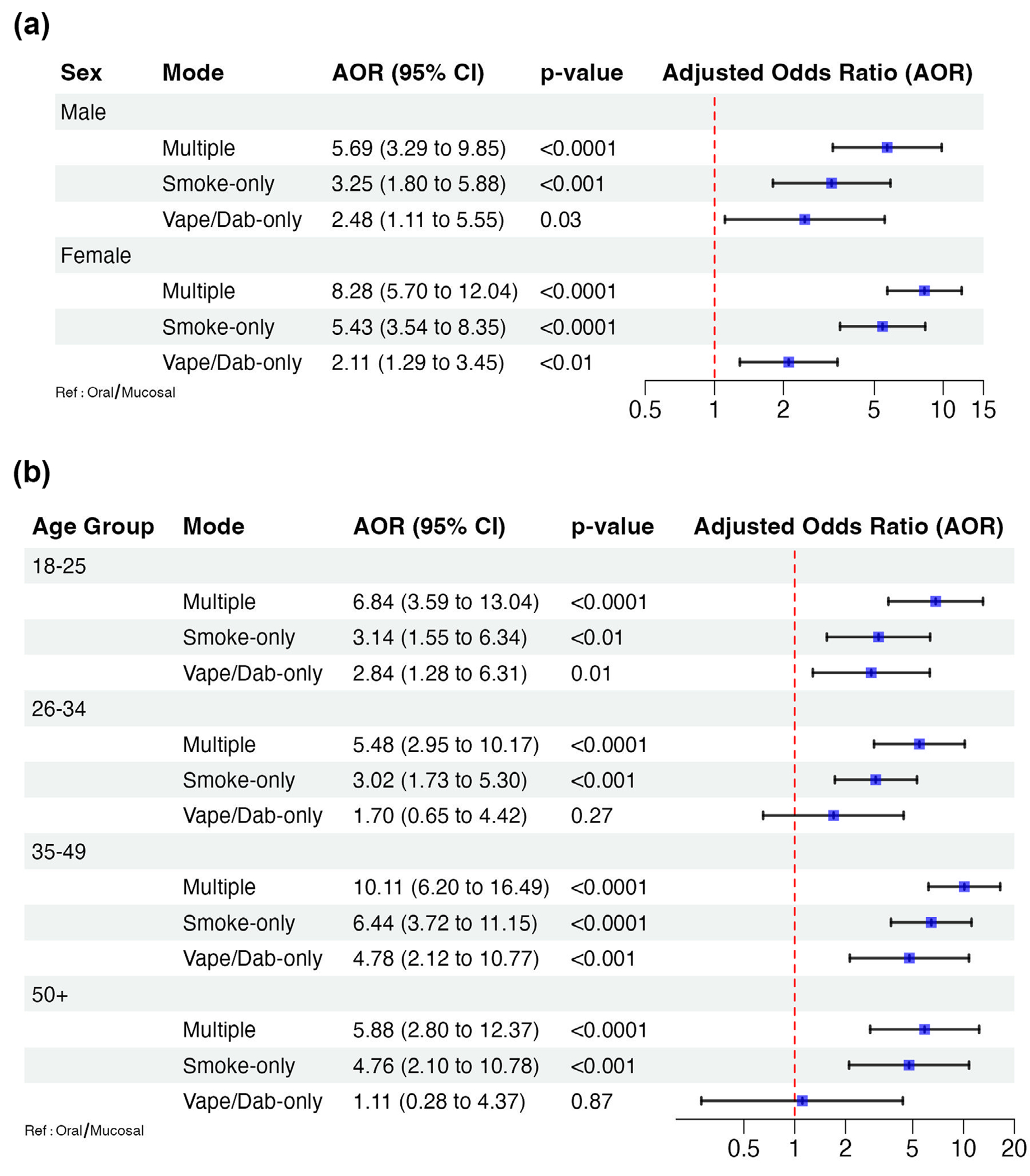
(a),(b) Methods of cannabis consumption and their association with cannabis use disorder: results from multi-variable logistic regression analysis among past-year cannabis users, 18 years and older (unweighted *n* = 25 549, weighted *n* = 58 850 309) 2022–2023 National Survey on Drug Use and Health (NSDUH), and stratified by sex and age. Stratified models adjusted for race/ethnicity, household income, education, health insurance, residential setting, state medical marijuana law status at the time of interview, age at cannabis initiation, motive for cannabis use, past-year days of use, past-year nicotine dependence, past-year alcohol use disorder, perceived risk of smoking cannabis, illicit drug use (excluding cannabis), past-year any mental illness and past-year psychotherapeutic use disorder. Age-stratified models additionally adjusted for sex; sex-stratified models additionally adjusted for age.

Given sex- and age-related differences in cannabis use preferences [28], we first tested effect modification by including multiplicative interaction terms (mode × sex; mode × age) in fully adjusted models (Table S3). In the absence of statistically significant interaction (*P* for joint test >0.05 for both), we report stratified estimates by sex and age for descriptive purposes and to facilitate interpretation. AORs and 95% CIs were reported [Figure 2(a,b)].

Next, to address potential confounding between mode of administration and frequency of cannabis use, we conducted stratified multi-variable logistic regression analyses by past-year cannabis use days, categorized as daily/near-daily (300–365 days) versus not daily/near-daily (<300 days) [25]. Within each stratum, we estimated AORs for CUD comparing mode-of-administration categories, using the same covariate set as in the primary model (Figure S2).

**FIGURE 3:**
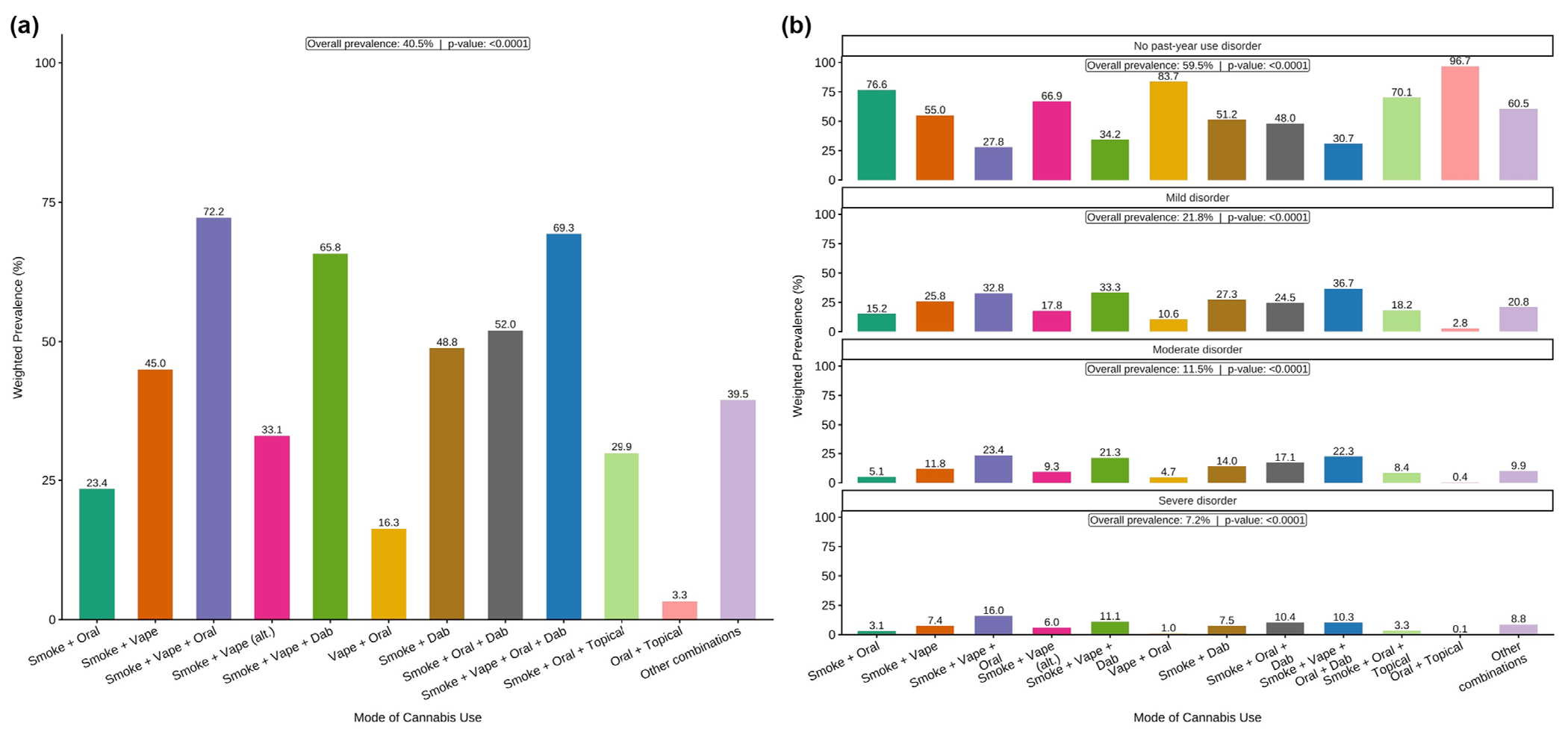
(a),(b). Cannabis use disorder prevalence and severity by method of cannabis among past-year cannabis users who self-reported cannabis use via multiple methods (≥2), 18 years and older (unweighted *n* = 15 144; weighted *n* = 31 713 769); 2022–2023 National Survey on Drug Use and Health (NSDUH). Oral = oral/mucosal *P-values are calculated using χ^2^ tests with Rao-Scott second-order correction ‘other combinations’ includes less frequent combinations of ≥2 methods (Please refer to Supplemental Table S1 for details).

**FIGURE 4:**
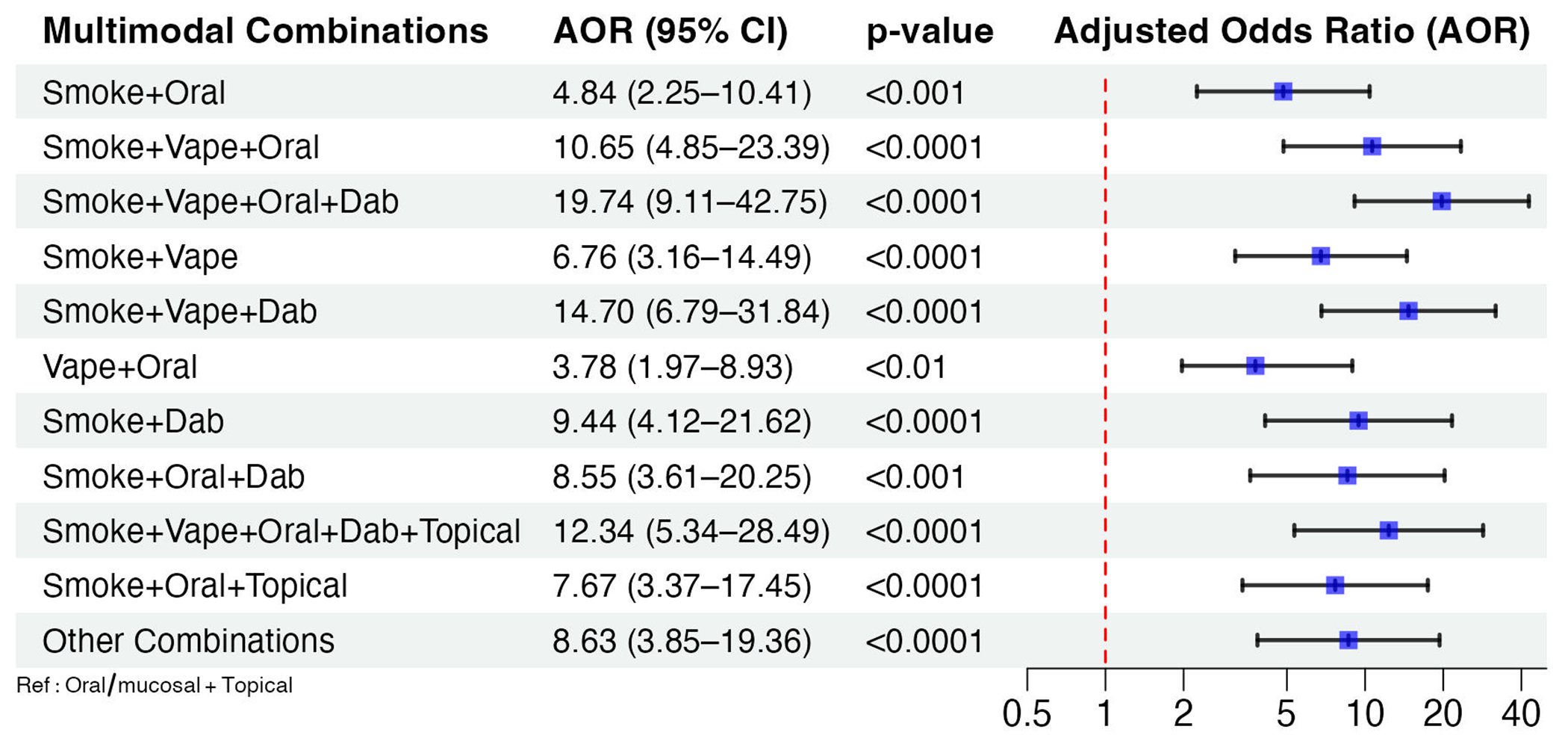
Multi-modal methods of cannabis consumption and their association with cannabis use disorder: results from multi-variable logistic regression analysis among past-year cannabis users who self-reported consuming cannabis using multiple methods (≥2), 18 years and older (unweighted *n* = 15 144; weighted *n* = 31 713 769); 2022–2023 National Survey on Drug Use and Health (NSDUH). Oral = oral/mucosal. Model adjusted for age, sex, race/ethnicity, household income, education, health insurance, residential setting, age at cannabis initiation, state medical marijuana law status at the time of interview, motive for cannabis use, past-year days of use, past-year nicotine dependence, past-year alcohol use disorder, perceived risk of smoking cannabis, illicit drug use (excluding cannabis), past-year any mental illness and past-year psychotherapeutic use disorder.

Additionally, we performed a subgroup analysis among multi-modal users only. Weighted prevalence estimates of CUD and severity across unique multi-modal combinations were reported [Figure 3(a,b)]. In analyses restricted to multi-modal users, vaping and dabbing were retained as distinct components and combinations were classified using the observed mode-pattern distribution. We reported results for the most frequent unique combinations and grouped less common patterns as ‘other combinations’ (Table S1). Multi-variable logistic regression models estimated AORs and 95% CIs for CUD, with ‘oral/mucosal + topicals’ as the reference (Figure 4). The model was adjusted for sociodemographic, behavioral, cannabis use and mental health covariates.

To limit the amount of missing data, multiple-imputed and recoded variables provided by NSDUH were used when available. Missing data across variables ranged from 0% to 0.73% (see Table S2 for details). All analyses were conducted in SAS 9.4 (SAS Institute). Inclusion of variables in the final models was guided by prior research, biological plausibility, univariate analyses and Type-3 analysis. The absence of multi-collinearity was confirmed by the variance inflation factor (VIF) values (all <2.5). All statistical tests were two-sided with an α level of <0.05. Analyses accounted for the NSDUH’s complex survey design. The primary research question and analysis plan for this study were not pre-registered on a publicly available platform, and accordingly, the findings should be considered exploratory.

## RESULTS

### Socio-demographic characteristics (Table 1)

All characteristics showed statistically significant differences across methods of use (*P* < 0.0001) (Table 1). Most respondents reported multi-modal cannabis use (53.9%). Younger adults (18–34 years) were more likely to report dab-only (37.3%) or multi-modal use (26.9%), whereas older adults (50+) predominated in smoke-only (38.0%) and topical-only (53.1%) groups. Men predominated across most modes except oral/mucosal and topical use. Non-Hispanic Whites comprised the majority overall (64.2%), while Non-Hispanic Blacks were overrepresented among smoke-only users (21.9%). Higher-income (≥$75 000) and college-educated adults (some college or more) were more common among oral/mucosal users (63.2% and 84.8%, respectively), whereas lower-income groups (≤$49 999) were concentrated among smoke-only users (54.2%). Most respondents lived in large metropolitan areas (56.5%), with rural residence more frequent among smoke-only (13.7%) and dab-only (16.6%) users. Most participants resided in states where a medical marijuana law had been enacted at the time of interview (78.1%).

### Cannabis use, behavioral and clinical characteristics by modes of use

Early initiation of cannabis use (<18 years) was reported by 57.9% overall, highest among multi-modal (62.2%) and smoke-only (61.0%) users (Table 2). Medical cannabis use was relatively uncommon (17.0%), but more frequent among topical-only (38.9%) users. Most participants used cannabis recreationally (83.0%), with medical-only use concentrated among topical users (31.6%). Use frequency varied sharply by mode, approximately one-third of multi-modal (32.6%) and dab-only (34.5%) users reported near-daily/daily use, compared with 7.5% of oral/mucosal-only users. Nicotine dependence was most common among smoke-only (26.0%) users and alcohol use disorder among multi-modal (27.2%) users. Perceived risk of harm from smoking cannabis 1 to 2 times per week was highest among topicals-only (68.2%) and oral/mucosal-only (67.6%) users and lowest among multi-mode (48.8%), dab-only (48.3%) and smoke-only (49.3%) users (vape-only: 59.4%, *P* < 0.0001). Illicit drug use (33.6%), any mental illness (45.1%) and psychotherapeutic use disorder (6.8%) were also the highest among multi-modal users.

### Cannabis use disorder and modes of use

Overall, 30.3% of the sample had CUD, with variation across modes (*P* < 0.0001) [Figure 1(a)]. The prevalence of CUD was highest among multi-modal users (40.5%), followed by dab-only (28.9%) and smoke-only users (24.3%). CUD was lower among oral/mucosal-only (4.4%) and topical-only (4.1%) users. Severity of CUD mirrored these differences [Figure 1(b)]. Moderate-to-severe CUD was concentrated among multi-modal users (11.5% and 7.2%, respectively) and dab-only users (10.5% and 7.1%, respectively), compared to less than 2% severe CUD among vape or oral/mucosal users. The majority of oral/mucosal-only (95.6%) and topical-only (95.9%) users had no CUD.

### Multi-variable logistic regression analyses of the overall sample

Compared to oral/mucosal-only users, odds of CUD were significantly higher for multi-modal (AOR = 4.14; 95% CI = 2.91–5.90), smoke-only (AOR = 2.98; 95% CI = 2.02–4.39), and vape/dab-only users (AOR = 1.89; 95% CI = 1.09–3.29) (Table 3).

CUD was independently associated with younger age, male sex and race/ethnicity: 18 to 25-year-olds had nearly fourfold higher odds of CUD than those 50 years or older (AOR = 3.90; 95% CI = 3.12–4.82), and males had greater odds than females (AOR = 1.41; 95% CI = 1.25–1.58). Hispanic and non-Hispanic Blacks had elevated odds of CUD relative to non-Hispanic Whites. Likewise, participants with a high school education had higher odds of CUD (AOR = 1.26; 95% CI = 1.05–1.50) than college graduates. Early cannabis initiation (<18 years) was associated with a greater odds (AOR = 1.32; 95% CI = 1.16–1.50) relative to later cannabis initiation.

Near-daily/daily cannabis use (300–365 days/year) was associated with higher odds of CUD (AOR = 6.68; 95% CI = 5.77–7.75) compared to non-near-daily/daily use. Nicotine dependence (AOR = 1.34; 95% CI = 1.09–1.66), alcohol use disorder (AOR = 1.53; 95% CI = 1.35–1.73), any illicit drug use (AOR = 1.45; 95% CI = 1.28–1.65), any mental illness (AOR = 2.43; 95% CI = 2.12–2.79) and psychotherapeutics use disorder (AOR = 1.80; 95% CI = 1.42–2.29) were independently associated with CUD.

### Stratified multi-variable logistic regression by sex and age

In sex-stratified adjusted models [Figure 2(a)] (reference: oral/mucosal-only), multi-mode use and smoke-only use were consistently associated with higher odds of CUD in both males [(AOR = 5.69; 95% CI = 3.29–9.85) and (AOR = 3.25; 95% CI = 1.80–5.88), respectively] and females [(AOR = 8.28; 95% CI = 5.70–12.04] and (AOR = 5.43; 95% CI = 3.54–8.35), respectively], whereas vape/dab-only use was also associated with increased odds in both sexes [(males: AOR = 2.48; 95% CI = 1.11–5.55); (females: AOR = 2.11; 95% CI = 1.29–3.45)].

Likewise, in age-stratified adjusted models [Figure 2(b)] (reference: oral/mucosal-only), multi-mode and smoke-only use were associated with higher odds of CUD across all age groups (AORs ranging from 5.48–10.11 and 3.02–6.44, respectively), whereas vape/dab-only use was associated with higher odds only among those 18 to 25 years old (AOR = 2.84; 95% CI = 1.28–6.31) and 35 to 49 years old (AOR = 4.78; 95% CI = 2.12–10.77). Tests for interaction indicated no significant effect modification of the route-CUD association by sex or age (*P* for joint test >0.05 for both) (Table S3).

### Stratified multi-variable logistic regression by days of cannabis use in the past year (near-daily/daily and non-near-daily/daily)

With oral/mucosal-only as reference, multi-mode (AOR = 6.27; 95% CI = 2.85–13.80), smoke-only (AOR = 4.15; 95% CI = 1.90–9.07) and vape/dab-only (AOR = 3.32; 95% CI = 1.36–8.15) use were each associated with higher odds of CUD among both near-daily/daily users and non–near-daily/daily users [(AOR = 6.60, 95% CI = 4.45–9.80); (AOR = 3.73; 95% CI = 2.45–5.67); and (AOR = 2.03; 95% CI = 1.13–3.67), respectively], indicating that the mode-CUD associations were consistent across frequency strata (Figure S2).

### Multi-modal methods of cannabis use and CUD

In the multi-modal subgroup, the overall prevalence of CUD was 40.5% [Figure 3(a)]. The highest prevalence was among those combining smoke + vape + oral/mucosal + dabbing (72.2%) and among those using smoke + vape + oral/mucosal + dab + topicals (69.3%). Conversely, prevalence was lower among oral/mucosal + topical (3.3%) or vape + oral/mucosal (16.3%).

Severity patterns paralleled these trends [Figure 3(b)]. Moderate-to-severe CUD affected 39.4% of smoke + vape + oral/mucosal + dab users, compared to oral/mucosal + topicals (0.5%) and vape + oral/mucosal (5.7%) users. Users of oral + topicals had the lowest prevalence of CUD (3.3%).

In regression analysis (Figure 4), compared to oral/mucosal + topicals users, the odds of CUD were significantly elevated among those using smoke + vape + oral/mucosal + dab (AOR = 19.74; 95% CI = 9.11–42.75), smoke + vape + dab (AOR = 14.70; 95% CI = 6.79–31.84), smoke + vape (AOR = 6.76; 95% CI = 3.16–14.49) and smoke + oral/mucosal (AOR = 4.84; 95% CI = 2.25–10.41). Even combinations lacking dabbing, such as vape + oral/mucosal (AOR = 3.78; 95% CI = 1.97–8.93), carried a significantly increased odds compared to non-inhaled forms.

## DISCUSSION

Our study found that the mode of cannabis use was strongly associated with both the prevalence and severity of CUD. With oral/mucosal-only as the reference, multi-modal use was associated with almost fourfold higher odds of CUD, while smoke-only and vape/dab-only conferred elevated odds, albeit to a lesser magnitude. The mode-CUD associations observed in the overall sample were consistent and statistically significant when stratified by age, sex and frequency of cannabis use. Combinations of multiple modes that involved smoking, vaping and dabbing were associated with the highest odds of CUD, exceeding 10-fold increases relative to the combination of oral/mucosal + topicals. Even less complex combinations, such as smoke + oral/mucosal or vape + oral/mucosal, carried significantly elevated risk, indicating that inhaled and high-potency modes of use may drive much of the observed burden of CUD. Furthermore, frequency of cannabis use, early initiation, co-occurring nicotine dependence, alcohol use disorder and mental health disorders were independently associated with CUD.

The strong association between multi-modal use and CUD aligns with a mechanistic interpretation in which combining inhaled (smoking, vaping/dabbing) [26, 29] with ingested routes (edibles) [30] may amplify cumulative psychoactive THC exposure and reinforcement by merging rapid-onset effects from inhalation with prolonged effects from ingestion [17–19]. Multi-modal use may also increase exposure to high-potency products such as concentrates [31], plausibly accelerating tolerance, intensifying withdrawal and fostering compulsive use. These findings are consistent with prior work linking multi-modal use [32] and frequent use to greater dependence [33–35], cannabis-related harms [34] and polysubstance involvement [34, 35]. Similarly, a prospective cohort of adolescents identified multi-modal use as a predictor of CUD [16]. Altogether, our findings highlight multi-modal use as a central behavioral marker of elevated CUD risk, underscoring the need to assess both frequency and diversity of cannabis consumption routes. An important point to note is that NSDUH captures mode of administration, but not cannabinoid composition (THC versus CBD), dose or formulation. This is especially relevant for vaping and oral/mucosal products, which may range from CBD-only to high-THC oils/concentrates, and for topicals, which are often CBD-dominant with little or no THC. Consequently, our oral/mucosal-only and oral/mucosal + topicals reference groups may include CBD-dominant products, potentially contributing to lower observed CUD burden. Sex and age-stratified analyses indicated that, relative to oral/mucosal-only use, multi-modal and smoke-only use were associated with elevated odds of CUD across strata. Although some strata showed larger point estimates (e.g. females versus males for multi-modal and smoke-only; and adults 35–49 years old for several modes), CIs overlapped, so these differences should not be interpreted as definitive between-group contrasts. Consistent with this, formal effect-modification tests in fully adjusted models did not provide evidence of statistically significant interaction, suggesting that the route-CUD associations were broadly similar by sex and age. Together, these findings support prevention and intervention messaging that extends beyond youth and remains relevant across demographic subgroups. Within the multi-modal subgroup, combinations involving dabbing had the highest burden of CUD compared to oral/mucosal + topicals and showed the steepest severity gradient. Dabbing-involving combinations, particularly those paired with both smoking and vaping, showed the strongest associations with CUD. Given that dabbing delivers concentrated extracts containing THC in concentrations of 70% and more [36, 37] and has been linked to psychosis, lung injury and cardiotoxicity [36, 38, 39], these findings underscore potential compounded risks when high-potency products are combined with multiple delivery methods.

Near-daily/daily users had more than sixfold greater odds of CUD compared to non-daily users, a pattern consistent with dependence models [40, 41]. Similarly, analyses stratified by frequency of cannabis use (near-daily/daily versus non–near-daily/daily) showed consistent mode-CUD associations in both strata, supporting that observed mode-related differences were not solely attributable to use frequency.

Co-occurring nicotine dependence, alcohol use disorder, other illicit drugs and past-year mental illness also predicted CUD independently, underscoring the role of polysubstance use and mental health comorbidity. Lack of perceived harm similarly tracked with greater risk [8, 42–45]. Additionally, we observed clear sociodemographic disparities, including higher risk at younger ages, early initiation and disproportionate burden among racial/ethnic minority groups. These disparities likely reflect structural and social determinants, such as greater exposure to unlicensed retail environments [46] rather than biological differences [47–49], highlighting critical targets for prevention and policy. Although DSM-5 does not specify a minimum frequency threshold, NSDUH assesses cannabis use disorder criteria primarily among respondents reporting cannabis use on 6 or more days in the past year (or with missing frequency) [50], and discordant cases because of imputation are uncommon. Frequency was a dominant correlate in our data: 62.8% of daily/near-daily users met criteria for CUD versus 18.9% of non-daily/near-daily users. Therefore, mode-of-use associations should be interpreted in the context of substantial differences in use frequency, underscoring the value of reporting both frequency and mode when characterizing CUD risk.

### Strengths and limitations

Strengths include the use of recent, nationally representative US data, detailed classification of cannabis use modes and comprehensive adjustment for covariates. Stratified analyses by sex, age and frequency further tested the robustness of CUD-mode associations. Limitations include the cross-sectional design, precluding causal inference and do not rule out reverse causality (e.g. individuals with emergent CUD shifting toward specific modes). All measures were self-reported, introducing potential recall or social desirability bias. Although we adjusted for a wide range of covariates, residual measurement limitations may affect our estimates. NSDUH does not capture THC potency, dose, product formulation, device characteristics or mode-specific dose/frequency, which could dilute or amplify mode-specific associations. Consequently, our combined vape/dab-only category likely groups heterogeneous exposures, introducing potential misclassification that may attenuate potency-related associations with CUD. In addition, CUD in NSDUH is a survey-based DSM-5 classification rather than a clinician-administered interview; criteria are assessed dichotomously, and severity reflects the number of criteria endorsed, not graded symptom intensity, which may introduce misclassification and limit nuance in interpreting severity. Our results suggest that the risks associated with cannabis use differ considerably by product type and route of administration, and therefore, regulators should consider a framework that classifies cannabis products into differential risk categories. For example, if cannabis was to be rescheduled, topical and edible products could be assigned to lower-risk tiers compared with inhaled products. In the context of expanding legalization and product diversity, the strong association between multi-modal use and CUD underscores the need for targeted prevention and harm-reduction efforts. Clinicians should screen not only for frequency, but also for modes and product types, while public health campaigns should highlight cumulative exposure risks from poly-route use and the disproportionate harms linked to concentrates. Policy measures could include potency labeling, product standardization and marketing restrictions that discourage multi-modal consumption.

## CONCLUSION

In conclusion, our findings underscore that CUD risk is shaped not only by frequency, but by the mode and complexity of use, with multi-modal and inhaled routes posing the highest risk. As cannabis markets evolve, integrating product-type differentiation into regulation, clinical screening and public health messaging will be essential to mitigate harm and guide safer patterns of use.

## Supplementary Material

add_70474-sup-0001-supplementalfigures1_4.16.26

add_70474-sup-0003-supplementaltables1_4.16.26

add_70474-sup-0002-supplementalfigures2_4.16.26

add_70474-sup-0004-supplementaltables2_4.16.26

add_70474-sup-0005-supplementaltables3_4.16.26

Additional supporting information can be found online in the Supporting Information section at the end of this article.

## Figures and Tables

**TABLE 1:** Socio-demographic characteristics of past-year cannabis users, 18 years and older (unweighted *n* = 25 549, weighted *n* = 58 850 309) by methods of cannabis consumption; 2022–2023 National Survey on Drug Use and Health.

Characteristics	Overall	Methods of cannabis use						*P*-value*
		Smoke-only	Vape-only	Oral/mucosal-only	Dab-only	Topicals-only	Multi-mode	
Unweighted *n*	25 549	6507	831	2775	143	149	15 144	
Weighted *n* weighted (%)	58 850 309	17 655 502 (30.0)	1 986 407 (3.4)	6 791 674 (11.5)	306 283 (0.5)	396 673 (0.7)	31 713 769 (53.9)	
Weighted proportion (%)								
Age groups (in years)								<0.0001
18–25	21.8	16.4	24.3	11.7	37.3	6.1	26.9	
26–34	24.1	20.2	25.3	21.0	26.7	10.8	26.9	
35–49	26.8	25.4	25.2	32.1	21.9	30.0	26.7	
50 or older	27.3	38.0	25.2	35.2	14.1	53.1	19.5	
Sex								<0.0001
Male	54.7	60.7	55.9	42.2	68.7	19.3	54.3	
Female	45.3	39.3	44.1	57.8	31.3	80.7	45.7	
Race/ethnicity								<0.0001
Non-Hispanic White	64.2	55.9	70.9	73.9	66.1	55.0	66.4	
Non-Hispanic Black	13.1	21.9	5.7	7.2	3.8	12.6	10.1	
Hispanic	15.6	15.8	16.5	11.7	26.9	23.5	16.0	
Non-Hispanic other	7.1	6.4	6.9	7.2	3.2	8.9	7.5	
Income (US$)								<0.0001
<20 000	17.2	24.0	13.6	6.5	15.1	11.7	16.1	
20 000–49 999	26.4	30.2	20.8	18.2	32.4	28.9	26.2	
50 000–74 999	14.6	13.9	14.8	12.1	12.0	15.6	15.5	
≥75 000	41.8	31.9	50.8	63.2	40.5	43.8	42.2	
Education								<0.0001
Less than high school	7.7	12.1	5.4	2.2	14.8	1.7	6.6	
High school	27.1	35.9	24.7	13.0	28.9	29.6	25.4	
Some college/associate degree	35.1	34.2	37.6	30.5	30.0	37.0	36.3	
College graduate or above	30.1	17.8	32.3	54.3	26.3	31.7	31.7	
Health insurance coverage								<0.0001
Yes	89.0	85.4	90.9	96.2	92.6	90.9	89.2	
No	11.0	14.6	9.1	3.8	7.4	9.1	10.8	
Residence (rurality)								<0.0001
Large metro	56.5	54.8	61.4	61.5	43.9	53.5	56.3	
Small metro	31.8	31.5	29.5	30.3	39.5	43.0	32.2	
Non-metro	11.7	13.7	9.1	8.2	16.6	3.5	11.5	
State medical marijuana law passed at the time of the interview								<0.001
In state where medical marijuana law passed before interview	78.1	74.8	84.1	79.6	89.1	85.4	79.1	
Not in state where medical marijuana law existed at time of interview	21.9	25.2	15.9	20.4	10.9	14.6	20.9	

*Note:* Multi-mode cannabis consumption means use of ≥ 2 methods.

Other-only modes (*n* = 29) were excluded from the analysis.

* *P*-values are calculated using χ^2^ tests with Rao-Scott second-order correction.

**TABLE 2:** Cannabis use, behavioral and clinical characteristics of past-year cannabis users, 18 years and older (unweighted *n* = 25 549, weighted *n* = 58 850 309) by methods of cannabis consumption; 2022–2023 National Survey on Drug Use and Health.

		Methods of cannabis use						
Characteristics	Overall	Smoke-only	Vape-only	Oral/mucosal-only	Dab-only	Topicals-only	Multi-mode	*P*-value*
Unweighted *n*	25 549	6507	831	2775	143	149	15 144	
Weighted *n* weighted (%)	58 850 309	17 655 502 (30.0)	1 986 407 (3.4)	6 791 674 (11.5)	306 283 (0.5)	396 673 (0.7)	31 713 769 (53.9)	
Weighted proportion (%)								
Cannabis first use age <18 years	57.9	61.0	45.7	34.9	57.5	28.8	62.2	<0.0001
Any medical cannabis use recommended by doctor (missing, *n* = 170)	17.0	14.4	14.8	13.9	21.5	38.9	18.9	<0.0001
All medical cannabis use recommended by doctor (missing, *n* = 179)	10.9	9.0	10.9	11.6	16.8	31.6	11.6	0.01
Cannabis use motive (missing, *n* = 179)								<0.0001
Medical-only	10.9	9.0	10.9	11.6	16.8	31.6	11.6	
Recreational-only	83.0	85.6	85.1	85.1	78.5	61.0	81.1	
Both medical and recreational	6.1	5.4	4.0	2.3	4.7	7.4	7.3	
Days cannabis was used in the past year								<0.0001
Near-daily/daily (300–365)	25.9	22.8	14.7	7.5	34.5	1.7	32.6	
Non-near-daily/daily (<300)	74.1	77.2	85.3	92.5	65.5	98.3	67.4	
Past-year nicotine dependence	17.5	26.0	10.3	4.8	18.3	7.8	16.1	<0.0001
Past-year alcohol use disorder	24.5	22.8	20.7	18.5	17.5	16.3	27.2	<0.0001
Perceived risk of self-harm, physically and in other ways, by smoking cannabis 1–2 times a week (missing, *n* = 187)								<0.0001
Yes	51.6	49.3	59.4	67.6	48.3	68.2	48.8	
No	48.4	50.7	40.6	32.4	51.7	31.8	51.3	
Past-year illicit drug use excluding cannabis	26.6	19.2	20.6	16.0	20.1	9.1	33.6	<0.0001
Past-year any mental illness	38.2	27.8	34.8	35.0	36.9	26.7	45.1	<0.0001
Past-year any psychotherapeutics use disorder	5.5	4.2	3.2	4.2	1.4	1.4	6.8	<0.0001

*Note*: Multi-mode cannabis consumption means use of ≥2 methods.

Other-only modes (*n* = 29) were excluded from the analysis.

* *P*-values are calculated using χ^2^ tests with Rao-Scott second-order correction.

**TABLE 3:** Methods of cannabis use and their association with cannabis use disorder: results from multi-variable logistic regression analysis among past-year cannabis users, 18 years and older (unweighted *n* = 25 549, weighted *n* = 58 850 309); 2022–2023 National Survey on Drug Use and Health.

Variables	OR (95% CI)	*P*-value*	AOR (95% CI)	*P*-value*
Modes of cannabis use				
Multiple	16.06 (11.23–22.96)	**<0.0001**	4.14 (2.91–5.90)	**<0.0001**
Smoke-only	7.62 (5.34–10.88)	**<0.0001**	2.98 (2.02–4.39)	**<0.0001**
Vape/dab-only	4.71 (2.72–8.17)	**<0.0001**	1.89 (1.09–3.29)	**0.02**
Oral/mucosal-only	**(Ref)**	–	**(Ref)**	–
Age groups (in years)				
18–25	3.55 (2.95–4.28)	**<0.0001**	3.90 (3.12–4.82)	**<0.0001**
26–34	2.35 (1.95–2.84)	**<0.0001**	1.98 (1.54–2.54)	**<0.0001**
35–49	1.77 (1.47–2.14)	**<0.0001**	1.47 (1.18–1.8)	**<0.001**
≥50	**(Ref)**	–	**(Ref)**	–
Sex				
Male	1.36 (1.24–1.50)	**<0.0001**	1.41 (1.25–1.58)	**<0.0001**
Female	**(Ref)**	–	**(Ref)**	–
Race ethnicity				
Hispanic	1.28 (1.13–1.45)	**<0.0001**	1.45 (1.22–1.71)	**<0.0001**
Non-Hispanic Black	1.32 (1.17–1.50)	**<0.0001**	1.41 (1.17–1.69)	**<0.001**
Non-Hispanic Other	1.33 (1.12–1.58)	**<0.0001**	1.73 (1.30–2.31)	**<0.001**
Non-Hispanic White	**(Ref)**	–	**(Ref)**	–
Income (US$)				
<20 000	**(Ref)**	–	**(Ref)**	–
20 000–49 999	**0.97 (0.86–1.09)**	0.63	0.90 (0.75–1.08)	0.27
50 000 –74 999	0.89 (0.76–1.05)	0.18	1.12 (0.91–1.37)	0.26
≥75 000	0.56 (0.50–0.63)	**<0.0001**	0.94 (0.77–1.15)	0.54
Education				
Less than high school	2.45 (2.13–2.82)	**<0.0001**	1.22 (0.99–1.51)	0.07
High school	2.17 (1.92–2.46)	**<0.0001**	1.26 (1.05–1.50)	**0.01**
Some college/associate degree	1.70 (1.49–1.91)	**<0.0001**	1.01 (0.82–1.23)	0.94
College graduate or above	**(Ref)**	–	**(Ref)**	–
Health insurance coverage				
Yes	0.69 (0.60–0.79)	**<0.0001**	1.04 (0.85–1.27)	0.78
No	**(Ref)**	–	**(Ref)**	–
Residence				
Large metro	0.86 (0.72–1.03)	0.10	1.00 (0.82–1.22)	0.94
Small metro	1.00 (0.83–1.21)	0.98	1.05 (0.85–1.30)	0.64
Non-metro			**(Ref)**	–
State marijuana law passed at the time of the interview				
In state where the medical marijuana law passed before interview	1.02 (0.99–1.12)	0.74	0.91 (0.78–1.07)	0.27
Not in state where medical marijuana law	**(Ref)**	–	**(Ref)**	–
Cannabis first use age <18				
Yes	2.16 (1.93–2.40)	<0.0001	1.32 (1.16–1.50)	**<0.0001**
No	**(Ref)**	–	**(Ref)**	–
Motive for cannabis use				
Recreational-only	0.78 (0.66–0.92)	**<0.01**	1.05 (0.87–1.27)	0.61
Both medical and recreational	1.41 (1.10–1.79)	**<0.01**	1.26 (0.92–1.73)	0.14
Medical only	**(Ref)**	–	**(Ref)**	–
Days cannabis was used (past-year)				
Near-daily/daily (300–365)	7.25 (6.40–8.20)	**<0.0001**	6.68 (5.77–7.75)	**<0.0001**
Non-near-daily/daily (<300)	**(Ref)**		**(Ref)**	–
Past-year nicotine dependence				
Yes	1.78 (1.56–2.03)	**<0.0001**	1.34 (1.09–1.66)	**<0.01**
No	**(Ref)**	–	**(Ref)**	–
Past-year alcohol use disorder				
Yes	1.79 (1.61–1.99)		1.53 (1.35–1.73)	**<0.0001**
No	**(Ref)**	–	**(Ref)**	–
Perceived risk of self-harm, physically and in other ways, by smoking cannabis 1–2 times a week				0.09
Yes	**(Ref)**	–	**(Ref)**	–
No	1.65 (1.52–1.79)	**<0.0001**	1.10 (0.98–1.23)	–
			**(Ref)**	–
Past-year Illicit drug use excluding cannabis				
Yes	2.51 (2.23–2.83)	**<0.0001**	1.45 (1.28–1.65)	**<0.0001**
No	**(Ref)**	–	**(Ref)**	
Past-year any mental illness				
Yes	2.69 (2.40–3.02)	**<0.0001**	2.43 (2.12–2.79)	**<0.0001**
No	**(Ref)**	–	**(Ref)**	
Past-year any psychotherapeutics use disorder				
Yes	2.70 (2.15–3.41)	**<0.0001**	1.80 (1.42–2.29)	**<0.0001**
No	**(Ref)**	–	**(Ref)**	–

*Note*: Multi-mode cannabis consumption means use of ≥2 methods; vape-only and dab-only were combined as a single category. Other modes (*n* = 29) and topicals only (*n* = 149) were excluded from the analysis.

Abbreviation: AOR, adjusted OR.

* Bolded *P*-values indicate a statistically significant association at α <0.05.

## Data Availability

The data used in this study are de-identified and publicly available for download from the SAMHSA website: https://www.samhsa.gov/data/.

## References

[1] Connor JP, Stjepanović D, Le Foll B, Hoch E, Budney AJ, Hall WD. Cannabis use and cannabis use disorder. Nat Rev Dis Primers. 2021;7(1):16. 10.1038/s41572-021-00247-4 33627670 PMC8655458

[2] Smart R, Pacula RL. Early evidence of the impact of cannabis legalization on cannabis use, cannabis use disorder, and the use of other substances: Findings from state policy evaluations. Am J Drug Alcohol Abuse. 2019;45(6):644–63. 10.1080/00952990.2019.1669626 31603710 PMC6934162

[3] Chapekis A, Shah S. Most Americans now live in a legal marijuana state—and most have at least one dispensary in their county Pew Research Center: Pew Research Center; 2024 [updated 2024, February 29; cited 2025 September 10]. Available from: https://www.pewresearch.org/short-reads/2024/02/29/most-americans-now-live-in-a-legal-marijuana-state-and-most-have-at-least-one-dispensary-in-their-county/

[4] Lapham GT, Matson TE, Bobb JF, Luce C, Oliver MM, Hamilton LK, Prevalence of cannabis use disorder and reasons for use among adults in a US state where recreational cannabis use is legal. JAMA Netw Open. 2023;6(8):e2328934–e. 10.1001/jamanetworkopen.2023.28934 37642968 PMC10466162

[5] Spindle TR, Bonn-Miller MO, Vandrey R. Changing landscape of cannabis: novel products, formulations, and methods of administration. Curr Opin Psychol. 2019;30:98–102. 10.1016/j.copsyc.2019.04.002 31071592 PMC7041884

[6] Leal WE, Moscrop-Blake K. The many forms of cannabis use: Prevalence and correlates of routes of administration among nationally representative samples of US adult and adolescent cannabis users. Addict Behav. 2024;159:108146. 10.1016/j.addbeh.2024.108146 39222559

[7] Lucas CJ, Galettis P, Schneider J. The pharmacokinetics and the pharmacodynamics of cannabinoids. Br J Clin Pharmacol. 2018;84(11):2477–82. 10.1111/bcp.13710 30001569 PMC6177698

[8] Choi NG, Marti CN, Choi BY. Associations between cannabis consumption methods and cannabis risk perception. IntJ Environ Res Public Health. 2024;21(8):986. 10.3390/ijerph21080986 39200597 PMC11353858

[9] Huestis MA. Human cannabinoid pharmacokinetics. Chem Biodivers. 2007;4(8):1770–804. 10.1002/cbdv.200790152 17712819 PMC2689518

[10] Grotenhermen F. Pharmacokinetics and pharmacodynamics of cannabinoids. Clin Pharmacokinet. 2003;42(4):327–60. 10.2165/00003088-200342040-00003 12648025

[11] Volkow ND, Blanco C. Substance use disorders: a comprehensive update of classification, epidemiology, neurobiology, clinical aspects, treatment and prevention. World Psychiatry. 2023;22(2):203–29. 10.1002/wps.21073 37159360 PMC10168177

[12] Bidwell LC, Martin-Willett R, Karoly HC. Advancing the science on cannabis concentrates and behavioural health. Drug Alcohol Rev. 2021;40(6):900–13. 10.1111/dar.13281 33783029 PMC9878551

[13] Compton WM, Han B, Jones CM, Blanco C. Cannabis use disorders among adults in the United States during a time of increasing use of cannabis. Drug Alcohol Depend. 2019;204:107468. 10.1016/j.drugalcdep.2019.05.008 31586809 PMC7028308

[14] Balodis I, MacKillop J. Cannabis use disorder. In: Recent Advances in Cannabinoid Research; 2018.

[15] Martin-Willett R, Elmore JS, Phillips PX, Bidwell LC. Meaningfully characterizing cannabis use for research and clinical settings: A comprehensive review of existing measures and proposed future directions. Psychiatry Clin Psychopharmacol. 2024;34(1):82–93. 10.5152/pcp.2024.23645 38883882 PMC11177636

[16] Walsh CA, Jafarzadeh N, Whaley RC, Han D-H, Leventhal A, Pedersen ER, Cannabis Products and Use Patterns Associated with Cannabis Use Disorder Symptoms Among Youth in Southern California. Cannabis. 2025;8(3):89–102. 10.26828/cannabis/2025/000323 41278419 PMC12640095

[17] Gunn RL, Aston ER, Sokolovsky AW, White HR, Jackson KM. Complex cannabis use patterns: Associations with cannabis consequences and cannabis use disorder symptomatology. Addict Behav. 2020;105:106329. 10.1016/j.addbeh.2020.106329 32044680 PMC7104573

[18] Simpson KA, Cho J, Barrington-Trimis JL. The association of type of cannabis product used and frequency of use with problematic cannabis use in a sample of young adult cannabis users. Drug Alcohol Depend. 2021;226:108865. 10.1016/j.drugalcdep.2021.108865 34216861 PMC8355167

[19] Ghafouri M, Correa da Costa S, Zare Dehnavi A, Gold MS, Rummans TA. Treatments for cannabis use disorder across the lifespan: a systematic review. Brain Sci. 2024;14(3):227. 10.3390/brainsci14030227 38539616 PMC10968391

[20] Levy NS, Mauro PM, Mauro CM, Segura LE, Martins SS. Joint perceptions of the risk and availability of cannabis in the United States, 2002-2018. Drug Alcohol Depend. 2021;226:108873. 10.1016/j.drugalcdep.2021.108873 34275699 PMC8478130

[21] [CBHSQ]. CfBHSaQ. 2022 National Survey on drug use and health public use file codebook. Rockville, MD: Substance Abuse and Mental Health Services Administration. 2023 [cited 2025 August 30]. Available from: https://www.datafiles.samhsa.gov/sites/default/files/field-uploads-protected/studies/NSDUH-2022/NSDUH-2022-datasets/NSDUH-2022-DS0001/NSDUH-2022-DS0001-info/NSDUH-2022-DS0001-info-codebook.pdf

[22] [CBHSQ] CfBHSaQ. National Survey on Drug Use and Health Public Use File Codebook; Substance Abuse and Mental Health Services Administration: Rockville, MD, USA, 2023 2022. Available from: https://www.datafiles.samhsa.gov/sites/default/files/field-uploads-protected/studies/NSDUH-2022/NSDUH-2022-datasets/NSDUH-2022-DS0001/NSDUH-2022-DS0001-info/NSDUH-2022-DS0001-info-codebook.pdf

[23] American Psychiatric Association. Diagnostic and Statistical Manual of Mental Disorders 5th ed. Washington: American Psychiatric Association; 2013.

[24] Hasin DS, O’brien CP, Auriacombe M, Borges G, Bucholz K, Budney A, DSM-5 criteria for substance use disorders: Recommendations and rationale. Am J Psychiatry. 2013;170(8):834–51. 10.1176/appi.ajp.2013.12060782 23903334 PMC3767415

[25] Mauro PM, Carliner H, Brown QL, Hasin DS, Shmulewitz D, Rahim-Juwel R, Age differences in daily and nondaily cannabis use in the United States, 2002–2014. J Stud Alcohol Drugs. 2018;79(3):423–31. 10.15288/jsad.2018.79.423 29885150 PMC6005250

[26] Spindle TR, Cone EJ, Schlienz NJ, Mitchell JM, Bigelow GE, Flegel R, Acute effects of smoked and vaporized cannabis in healthy adults who infrequently use cannabis: A crossover trial. JAMA Netw Open. 2018;1(7):e184841–e. 10.1001/jamanetworkopen.2018.4841 30646391 PMC6324384

[27] Meehan-Atrash J, Luo W, McWhirter KJ, Strongin RM. Aerosol gas-phase components from cannabis e-cigarettes and dabbing: Mechanistic insight and quantitative risk analysis. ACS Omega. 2019;4(14):16111–20. 10.1021/acsomega.9b02301 31592479 PMC6777088

[28] Cuttler C, Mischley LK, Sexton M. Sex differences in cannabis use and effects: A cross-sectional survey of cannabis users. Cannabis Cannabinoid Res. 2016;1(1):166–75. 10.1089/can.2016.0010 28861492 PMC5576608

[29] Newmeyer MN, Swortwood MJ, Abulseoud OA, Huestis MA. Subjective and physiological effects, and expired carbon monoxide concentrations in frequent and occasional cannabis smokers following smoked, vaporized, and oral cannabis administration. Drug Alcohol Depend. 2017;175:67–76. 10.1016/j.drugalcdep.2017.02.003 28407543

[30] Behzad D, Patel S, Besa R, Chan AWH, Chen S, Rueda S, Effects of different methods of cannabis use on cognition and blood THC: A systematic review. Prog Neuro-Psychopharmacol Biol Psych. 2025;139:111399. 10.1016/j.pnpbp.2025.111399 40368229

[31] Chen MY, Brooks-Russell A, Bryan AD, Bidwell LC. Mode matters: exploring how modes of cannabis administration affect THC plasma concentrations and subjective effects. J Cannabis Res. 2025;7(1):28. 10.1186/s42238-025-00282-y 40410918 PMC12100805

[32] Kim N, Flora S, Macander CE. Multi-modal cannabis use among US young adults: Findings from the 2022 and 2023 BRFSS in 23 states. Int J Environ Res Public Health. 2025;22(4):495. 10.3390/ijerph22040495 40283724 PMC12026715

[33] Cloutier RM, Calhoun BH, Linden-Carmichael AN. Associations of mode of administration on cannabis consumption and subjective intoxication in daily life. Psychol Addict Behav. 2022;36(1):67–77. 10.1037/adb0000726 34472879 PMC8831393

[34] Swan C, Ferro MA, Thompson K. Does how you use matter? The link between mode of use and cannabis-related risk. Addict Behav. 2021;112:106620. 10.1016/j.addbeh.2020.106620 32911353

[35] Baggio S, Deline S, Studer J, Mohler-Kuo M, Daeppen J-B, Gmel G. Routes of administration of cannabis used for nonmedical purposes and associations with patterns of drug use. J Adolesc Health. 2014;54(2):235–40. 10.1016/j.jadohealth.2013.08.013 24119417

[36] Rossi G, Beck M. A little dab will do: A case of cannabis-induced psychosis. Cureus. 2020;12(9):e10311. 10.7759/cureus.10311 33052273 PMC7544610

[37] Stogner JM, Miller BL. Assessing the dangers of “dabbing”: mere marijuana or harmful new trend? Pediatrics. 2015;136(1):1–3. 10.1542/peds.2015-0454 26077476

[38] Stephens D, Patel JK, Angelo D, Frunzi J. Cannabis butane hash oil dabbing induced lung injury mimicking atypical pneumonia. Cureus. 2020;12(2):e7033. 10.7759/cureus.7033 32211266 PMC7082782

[39] Alzghari SK, Fung V, Rickner SS, Chacko L, Fleming SW, F-Abft SFMS. To dab or not to dab: Rising concerns regarding the toxicity of cannabis concentrates. Cureus. 2017;9(9):e1676. 29152433 10.7759/cureus.1676 PMC5679763

[40] Robinson T, Ali MU, Easterbrook B, Coronado-Montoya S, Daldegan-Bueno D, Hall W, Identifying risk-thresholds for the association between frequency of cannabis use and development of cannabis use disorder: A systematic review and meta-analysis. Drug Alcohol Depend. 2022;238:109582. 10.1016/j.drugalcdep.2022.109582 35932748

[41] Leung J, Chan GCK, Hides L, Hall WD. What is the prevalence and risk of cannabis use disorders among people who use cannabis? A systematic review and meta-analysis. Addict Behav. 2020;109:106479. 10.1016/j.addbeh.2020.106479 32485547

[42] Ferdinand RF, Sondeijker F, Van Der Ende J, Selten JP, Huizink A, Verhulst FC. Cannabis use predicts future psychotic symptoms, and vice versa. Addiction. 2005;100(5):612–8. 10.1111/j.1360-0443.2005.01070.x 15847618

[43] Womack SR, Shaw DS, Weaver CM, Forbes EE. Bidirectional associations between cannabis use and depressive symptoms from adolescence through early adulthood among at-risk young men. J Stud Alcohol Drugs. 2016;77(2):287–97. 10.15288/jsad.2016.77.287 26997187 PMC4803661

[44] Radhakrishnan R, Pries L-K, Erzin G, Ten Have M, de Graaf R, van Dorsselaer S, Bidirectional relationships between cannabis use, anxiety and depressive symptoms in the mediation of the association with psychotic experience: further support for an affective pathway to psychosis. Psychol Med. 2023;53(12):5551–7. 10.1017/S0033291722002756 36093677 PMC10482707

[45] Mennis J, McKeon TP, Stahler GJ. Recreational cannabis legalization alters associations among cannabis use, perception of risk, and cannabis use disorder treatment for adolescents and young adults. Addict Behav. 2023;138:107552. 10.1016/j.addbeh.2022.107552 36413909

[46] Unger JB, Vos RO, Wu JS, Hardaway K, Sarain AYL, Soto DW, Locations of licensed and unlicensed cannabis retailers in California: A threat to health equity? Prev Med Rep. 2020;19:101165. 10.1016/j.pmedr.2020.101165 32714779 PMC7378688

[47] Wu L-T, Zhu H, Swartz MS. Trends in cannabis use disorders among racial/ethnic population groups in the United States. Drug Alcohol Depend. 2016;165:181–90. 10.1016/j.drugalcdep.2016.06.002 27317045 PMC4939114

[48] Wu L-T, Brady KT, Mannelli P, Killeen TK, Workgroup NA. Cannabis use disorders are comparatively prevalent among nonwhite racial/ethnic groups and adolescents: A national study. J Psychiatr Res. 2014;50:26–35. 10.1016/j.jpsychires.2013.11.010 24342767 PMC3941308

[49] Montgomery L, Dixon S, Mantey DS. Racial and ethnic differences in cannabis use and cannabis use disorder: Implications for researchers. Curr Addict Rep. 2022;9(1):14–22. 10.1007/s40429-021-00404-5 35251891 PMC8896813

[50] 2023 National Survey on Drug Use and Health (NSDUH): Methodological summary and definitions. Center for Behavioral Health Statistics and Quality: Substance Abuse and Mental Health Services Administration. 2023 [cited 2024 February 15]. Available from: https://www.samhsa.gov/data/report/2023-methodological-summary-and-definitions

