# Supplementary figures and images for: Associations between modes of cannabis use and cannabis use disorder: Evidence from the 2022 to 2023 United States National Survey on Drug Use and Health
Source: Addiction. Author manuscript; Available in PMC 2026 Jul 23. (PMC13395235; doi:10.1111/add.70474)

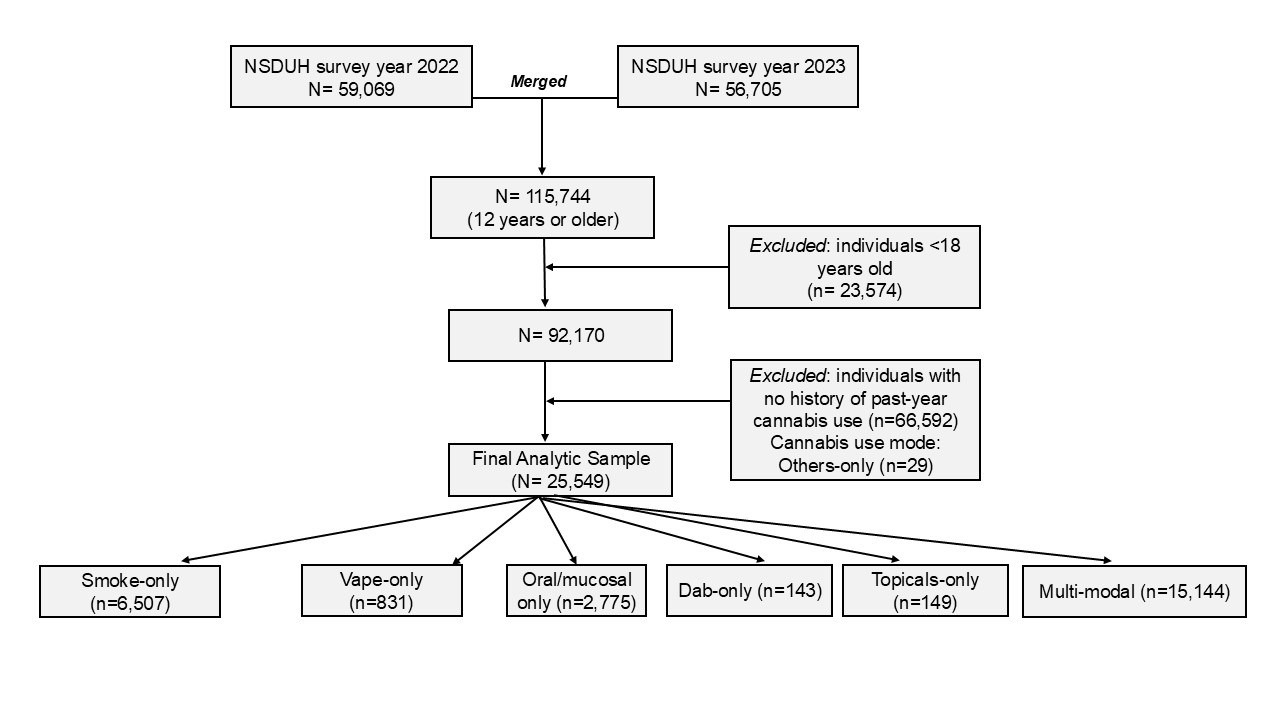

Supplement: add_70474-sup-0001-supplementalfigures1_4.16.26 [file NIHMS2193610-supplement-add_70474-sup-0001-supplementalfigures1_4_16_26.jpg]

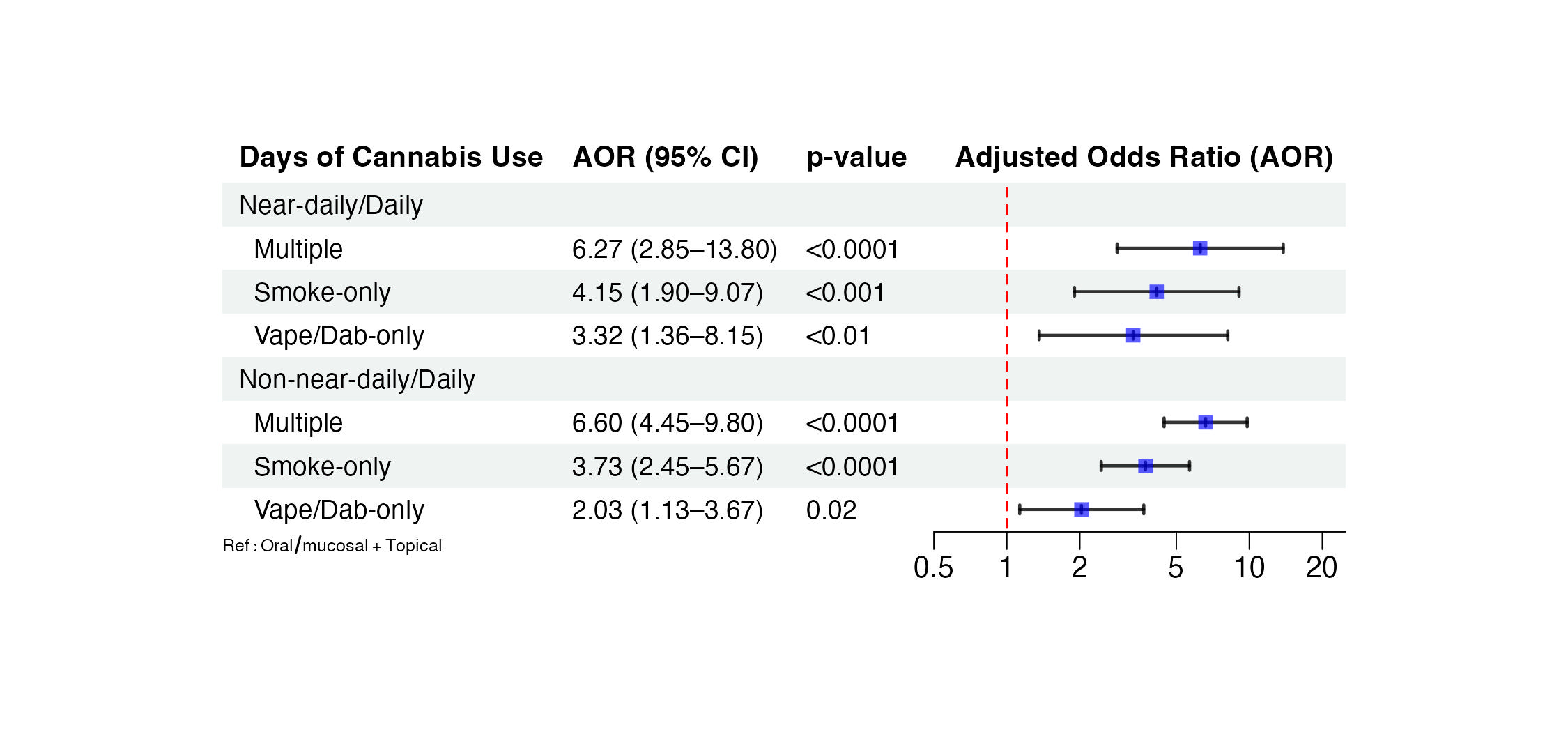

Supplement: add_70474-sup-0002-supplementalfigures2_4.16.26 [file NIHMS2193610-supplement-add_70474-sup-0002-supplementalfigures2_4_16_26.jpeg]
