# Supplementary material for: Associations between modes of cannabis use and cannabis use disorder: Evidence from the 2022 to 2023 United States National Survey on Drug Use and Health
Source: Addiction. Author manuscript; Available in PMC 2026 Jul 23. (PMC13395235; doi:10.1111/add.70474)
Supplement: add_70474-sup-0003-supplementaltables1_4.16.26 [file NIHMS2193610-supplement-add_70474-sup-0003-supplementaltables1_4_16_26.docx]

**Supplemental Table S1**. Detailed Frequency of Self-reported Modes of Cannabis Use Among Past-year Cannabis Users, 18 years and Older, 2022-2023, National Survey on Drug Use and Health

| **Modes of Cannabis Use** | **Frequency** | **Percent** | **Cumulative** **Frequency** | **Cumulative** **Percent** |
| --- | --- | --- | --- | --- |
| **Smoke** | 6507 | 25.44 | 6507 | 25.44 |
| **Smoke+Oral** | 3019 | 11.80 | 9526 | 37.24 |
| **Oral** | 2775 | 10.85 | 12301 | 48.09 |
| **Smoke+Vape+Oral** | 2524 | 9.87 | 14825 | 57.96 |
| **Smoke+Vape+Oral+Dab** | 2096 | 8.19 | 16921 | 66.15 |
| **Smoke+Vape** | 2030 | 7.94 | 18951 | 74.09 |
| **Smoke+Vape+Dab** | 986 | 3.85 | 19937 | 77.95 |
| **Vape** | 831 | 3.25 | 20768 | 81.19 |
| **Vape+Oral** | 667 | 2.61 | 21435 | 83.80 |
| **Smoke+Dab** | 627 | 2.45 | 22062 | 86.25 |
| **Smoke+Oral+Dab** | 545 | 2.13 | 22607 | 88.38 |
| **Smoke+Vape+Oral+Dab+Topical** | 413 | 1.61 | 23020 | 90.00 |
| **Smoke+Oral+Topical** | 326 | 1.27 | 23346 | 91.27 |
| **Oral+Topical** | 286 | 1.12 | 23632 | 92.39 |
| **Smoke+Vape+Oral+Topical** | 277 | 1.08 | 23909 | 93.47 |
| **Smoke+Topical** | 217 | 0.85 | 24126 | 94.32 |
| **Topical** | 149 | 0.58 | 24275 | 94.91 |
| **Dab** | 143 | 0.56 | 24418 | 95.46 |
| **Vape+Oral+Topical** | 100 | 0.39 | 24518 | 95.86 |
| **Smoke+Vape+Topical** | 98 | 0.38 | 24616 | 96.24 |
| **Smoke+Oral+Dab+Topical** | 90 | 0.35 | 24706 | 96.59 |
| **Smoke+Vape+Oral+Dab+Topical+Other** | 83 | 0.32 | 24789 | 96.92 |
| **Oral+Other** | 79 | 0.31 | 24868 | 97.22 |
| **Smoke+Vape+Oral+Dab+Other** | 74 | 0.29 | 24942 | 97.51 |
| **Smoke+Vape+Dab+Topical** | 66 | 0.26 | 25008 | 97.77 |
| **Vape+Dab** | 63 | 0.25 | 25071 | 98.02 |
| **Vape+Oral+Dab** | 58 | 0.23 | 25129 | 98.24 |
| **Oral+Dab** | 55 | 0.22 | 25184 | 98.46 |
| **Smoke+Other** | 53 | 0.21 | 25237 | 98.67 |
| **Smoke+Vape+Oral+Other** | 47 | 0.18 | 25284 | 98.85 |
| **Smoke+Oral+Other** | 43 | 0.17 | 25327 | 99.02 |
| **Smoke+Dab+Topical** | 41 | 0.16 | 25368 | 99.18 |
| **Vape+Topical** | 34 | 0.13 | 25402 | 99.31 |
| **Other** | 29 | 0.11 | 25431 | 99.43 |
| **Smoke+Vape+Dab+Other** | 20 | 0.08 | 25451 | 99.50 |
| **Smoke+Vape+Oral+Topical+Other** | 16 | 0.06 | 25467 | 99.57 |
| **Oral+Dab+Topical** | 12 | 0.05 | 25479 | 99.61 |
| **Smoke+Oral+Dab+Other** | 12 | 0.05 | 25491 | 99.66 |
| **Smoke+Vape+Other** | 10 | 0.04 | 25501 | 99.70 |
| **Vape+Oral+Dab+Topical** | 10 | 0.04 | 25511 | 99.74 |
| **Oral+Topical+Other** | 9 | 0.04 | 25520 | 99.77 |
| **Smoke+Dab+Other** | 8 | 0.03 | 25528 | 99.80 |
| **Dab+Topical** | 7 | 0.03 | 25535 | 99.83 |
| **Smoke+Topical+Other** | 6 | 0.02 | 25541 | 99.86 |
| **Vape+Dab+Topical** | 6 | 0.02 | 25547 | 99.88 |
| **Vape+Oral+Other** | 6 | 0.02 | 25553 | 99.90 |
| **Topical+Other** | 5 | 0.02 | 25558 | 99.92 |
| **Smoke+Oral+Dab+Topical+Other** | 4 | 0.02 | 25562 | 99.94 |
| **Smoke+Oral+Topical+Other** | 4 | 0.02 | 25566 | 99.95 |
| **Dab+Other** | 3 | 0.01 | 25569 | 99.96 |
| **Oral+Dab+Other** | 2 | 0.01 | 25571 | 99.97 |
| **Smoke+Vape+Topical+Other** | 2 | 0.01 | 25573 | 99.98 |
| **Vape+Oral+Topical+Other** | 2 | 0.01 | 25575 | 99.99 |
| **Smoke+Dab+Topical+Other** | 1 | 0.00 | 25576 | 99.99 |
| **Vape+Oral+Dab+Topical+Other** | 1 | 0.00 | 25577 | 100.00 |
| **Vape+Other** | 1 | 0.00 | 25578 | 100.00 |
| **Notes**: Oral= Oral/Mucosal and included eating or drinking, drops/strips/lozenges/sprays in the mouth, and pills | | | | |
