# Supplementary material for: Associations between modes of cannabis use and cannabis use disorder: Evidence from the 2022 to 2023 United States National Survey on Drug Use and Health
Source: Addiction. Author manuscript; Available in PMC 2026 Jul 23. (PMC13395235; doi:10.1111/add.70474)
Supplement: add_70474-sup-0004-supplementaltables2_4.16.26 [file NIHMS2193610-supplement-add_70474-sup-0004-supplementaltables2_4_16_26.docx]

**Supplemental Table S2.** Details on the Operationalization of Variables Used in the Analysis and Missing Data Information

| **Variables** | **Variable name in NSDUH:** | **Defined as/Label:** | **Coded in the NSDUH codebook as:** | **Re-coded in our analysis as:** | **Missing data (%)** |
| --- | --- | --- | --- | --- | --- |
| Age | CATAG3 | RC-AGE CATEGORY RECODE (5-LEVELS) | 1 = 12-17 Years Old  2= 18-25 Years Old  3= 26-34 Years Old  4= 35-49 Years old  5= 50 or Older | NA  (Sample restricted to 18 years and older) | 0 |
| Sex | IRSEX | IMPUTATION REVISED GENDER | 1= Male  2= Female | NA | 0 |
| Race/ethnicity | NEWRACE2 | RC-RACE/HISPANICITY RECODE (7 LEVELS) | 1= Non-Hispanic White  2= Non-Hispanic White Black/African American  3= Non-Hispanic Native American/AK Native  4= Non-Hispanic Native HI/Other Pacific Islanders  5= Non-Hispanic Asian  6= Non-Hispanic more than one race  7= Hispanic | 1= Non-Hispanic White  2= Non-Hispanic Black/African American  3= Hispanic  4= Non-Hispanic Others (Included Non-Hispanic Native American/AK Native, Non-Hispanic Asian, and Non-Hispanic more than one race) | 0 |
| Family Income | INCOME | RC-TOTAL FAMILY INCOME | 1= Less than $ 20,000  2= $20,000-$49,999  3= $50,000-$74,999  4= $75,000 or More | NA | 0 |
| Education | IREDUHIGHST2 | EDUCATION-RECODED IMPUTATION REVISED | 1 = Fifth grade or less grade completed  2 = Sixth grade completed  3 = Seventh grade completed  4 = Eighth grade completed  5 = Ninth grade completed  6 = Tenth grade completed  7 = Eleventh or Twelfth grade completed, no diploma  8 = High school diploma/GED  9 = Some college credit, but no degree  10 = Associate's degree (for example, AA, AS)  11 = College graduate or higher | 1= < High School  2= High School  3= Some college/Associate degree  4= College graduate and above | 0 |
| Health insurance coverage | IRINSUR4 | Having any health insurance  RC-OVERALL HEALTH INSURANCE - IMPUTATION REVISED | 1 = Yes, respondent is covered by health insurance  2 = No, respondent is not covered by health insurance | NA | 0 |
| Residence (rurality) status | COUTYP4 | Based on the 2013 Rural/Urban Continuum Codes.  COUNTY METRO/NONMETRO STATUS (2013 3-LEVEL | 1= Large Metro  2= Small Metro  3= Nonmetro | NA | 0 |
| The state medical marijuana law was passed at the time of the interview | MEDMJPA2 | Indicates whether respondents were living in a State in which a law allowing the use of marijuana for medical reasons had been  passed at the time of the interview.  STATE MEDICAL MJ LAW PASSED AT TIME OF INTERVIEW | 1 = In State where medical MJ law passed before interview  2 = Not In State where med MJ law existed at time of interview | NA | 0 |
| Past-year cannabis use | MRJYR | RC- MARIJUANA -PAST YEAR USE | 0 = Did not use in the past year  1 = Used within the past year | NA | 0 |
| Past-year cannabis use by smoking | IRMJYRSMOKE | MARIJUANA SMOKING PAST YEAR- IMPUTATION REVISED | 1= Within the past year  2= Did not smoke marijuana in the past year  9= Did not use marijuana in the past 12 months | NA | 0 |
| Past-year cannabis use by vaping | IRMJYRVAPE | MARIJUANA VAPING PAST YEAR - IMPUTATION REVISED | 1= Within the past 12 months  2 = Did not vape marijuana in the past 12 months  9 = Did not use marijuana in the past 12 months | NA | 0 |
| Past-year cannabis use by dabbing | IRMJYRDAB | MARIJUANA DABBING WAXES, SHATTER, OR CONC PAST YEAR-IMP REV | 1 = Within the past 12 months  2 = Did not dab marijuana in the past 12 months  9 = Did not use marijuana in the past 12 months | NA | 0 |
| Past-year cannabis use eating/drinking | IRMJYREAT | MARIJUANA EATING OR DRINKING PAST YEAR - IMPUTATION REVISED | 1 = Within the past 12 months  2 = Did not eat marijuana in the past 12 months  9 = Did not use marijuana in the past 12 months | NA | 0 |
| Past-year cannabis use with mouth by drops, strips, lozenges, sprays in the mouth | IRMJYRMOUTH | MARIJUANA DROPS/STRIPS/LOZENGS/SPARYS IN MOUTH PAST YR - IMP REV | 1 = Within the past 12 months  2 = Did not dissolve marijuana in the mouth in the past 12 months  9 = Did not use marijuana in the past 12 months | NA | 0 |
| Past-year cannabis use as pills | IRMJYRPILL | MARIJUANA PILLS PAST YEAR - IMPUTATION REVISED | 1 = Within the past 12 months  2 = Did not take marijuana pills in the past 12 months  9 = Did not use marijuana in the past 12 months | NA | 0 |
| Past-year cannabis use as topicals | IRMJYRSKIN | MARIJUANA LOTION/CREAM/PATCH TO SKIN PAST YEAR - IMP REV | 1 = Within the past 12 months  2 = Did not use marijuana lotion in the past 12 months  9 = Did not use marijuana in the past 12 months | NA | 0 |
| Past-year cannabis use by other way/modes | IRMJYROTHER | MARIJUANA OTHER WAY PAST YEAR - IMPUTATION REVISED | 1= Within the past 12 months  2 = Did not use marijuana in any other way in the past 12 months  9 = Did not use marijuana in the past 12 months | NA | 0 |
| Cannabis first use age (<18 years) | FUMJ18 | RC-FIRST USED MARIJUANA PRIOR TO AGE 18 | 1 = Yes (IRMJAGE<18)  2 = No (IRMJAGE>=18) | NA | 0 |
| Any medical cannabis use recommended by a doctor | MJANYMEDYR | Earlier, you reported using marijuana or any cannabis product in the past year. Was any of your marijuana or cannabis product use in the past 12  months recommended by a doctor or other health care professional? | 1 = Yes  2 = No  91 = NEVER USED MARIJUANA  93 = DID NOT USE MARIJUANA IN THE PAST 12 MONTHS  94 = DON'T KNOW  97 = REFUSED  98= Blank (NO ANSWER) | NA | 0.66 |
| All medical cannabis use recommended by the doctor | MJALLMEDYR | Was all of your marijuana or cannabis use in the past 12 months recommended by a doctor or other health care professional? | 1 = Yes  2 = No  85 = BAD DATA Logically Assigned  91 = NEVER USED MARIJUANA  93 = DID NOT USE MARIJUANA IN THE PAST 12 MOS  94 = DON'T KNOW  97 = REFUSED  98 = BLANK (NO ANSWER)  99 = LEGITIMATE SKIP | NA | 0.70 |
| Cannabis use motive | NA (We created this variable) using MJANYMEDYR AND MJALLMEDYR | Motive for cannabis use | 1= Medical only  2= Both medical and recreational  3= Recreational-only | IF MJALLMEDYR=1 then cannabis use motive=Medical only.  If MJANYMEDYR=1 and MJALLMEDYR=0 then motive = Both  If MJANYMEDYR=0, then motive = Recreational only | 0.70 |
| Days cannabis was used in the past year | MRJYDAYS | The number of days that marijuana was used in the  past month among past month marijuana users.  RC-# OF DAYS USED MARIJUANA IN PAST YEAR | 1 = 1-11 Days (IRMJFY=1-11)  2 = 12-49 Days (IRMJFY=12-49)  3 = 50-99 Days (IRMJFY=50-99)  4 = 100-299 Days (IRMJFY=100-299)  5 = 300-365 Days (IRMJFY=300-365) | 1= Non-Near daily/daily users (those reported using <300 days)  2= Near daily/daily users (those reported using 300-365 days) | 0 |
| Past-month nicotine dependence | IRPMNICDEP | NICOTINE DEPENDENCE IN PAST MONTH - IMPUTATION REVISED: Imputation-revised version of the nicotine dependence variable (PMNICDEP) derived  from the Nicotine Dependence Syndrome Scale (NDSS) and the Fagerstrom Test of Nicotine Dependence (FTND). Based on the NDSS and the  FTND, a respondent who smoked cigarettes in the past month, was defined as having nicotine dependence if they met either the NDSS or the FTND  criteria for dependence. | 0= No  1= Yes | NA | 0 |
| Past-year alcohol use disorder | IRPYUD5ALC | Alcohol use disorder based on the criteria in the Diagnostic and Statistical Manual of Mental Disorders, 5th edition  ALCOHOL USE DISORDER IN THE PAST YEAR - IMP REV | 0= No  1= Yes | NA | 0 |
| Perceived risk of self-harm, physically and in other ways, by smoking cannabis 1-2 times a week | RSKMRJWK | How much do people risk harming themselves physically and in other ways when they smoke marijuana once or twice a week? | 1 = No risk  2 = Slight risk  3 = Moderate risk  4 = Great risk  85 = BAD DATA Logically assigned  94 = DON'T KNOW  97 = REFUSED  98 = BLANK (NO ANSWER) | 0= No  1= Yes (Slight, moderate, and great risk)  Missing= (85, 94, 97, 98) | 0.73 |
| Past-year illicit drug use excluding cannabis | ILLEMYR | RC-ILLICIT DRUG OTHER THAN MARIJUANA - PAST YEAR | 0 = Never used drug/used only marijuana past year  1 = Illicit drug, except for marijuana used past year | NA | 0 |
| Past-year any mental illness | AMIPY | Recoded and Imputed variable provided by NSDUH  Indicator for Any Mental Illness (AMI) based on the 2012 predicted probability of SMI (SMIPPPY). If  SMIPPPY is greater than or equal to a specified cutoff point (0.0192519810), then AMIPY=1, and if SMIPPPY is less than the cutoff point, then AMIPY=0. AMI is defined as having Serious, Moderate, or Mild Mental Illness | 0 = No Past Yr Any Mental Illness 1 = Past Year Any Mental Illness | NA | 0 |
| Past-year any psychotherapeutic use disorder | UD5PSYANY | Recoded variable provided by NSDUH. UD5PSYANY was created using the DSM-5-based SUD variables that include use disorder data from all past year users of prescription drugs. | 0 = No (all source variables = 0)  1 = Yes (any source variable = 1) | NA | 0 |
| **Note:** RC- recoded by NSDUH and provided for analysis; MJ- marijuana (cannabis) | | | | | |
