# Supplementary material for: Associations between modes of cannabis use and cannabis use disorder: Evidence from the 2022 to 2023 United States National Survey on Drug Use and Health
Source: Addiction. Author manuscript; Available in PMC 2026 Jul 23. (PMC13395235; doi:10.1111/add.70474)
Supplement: add_70474-sup-0005-supplementaltables3_4.16.26 [file NIHMS2193610-supplement-add_70474-sup-0005-supplementaltables3_4_16_26.docx]

**Supplemental Table S3.** Joint interaction tests and interaction-term estimates (multiplicative scale) Between Modes of Cannabis Use and Age and Sex

| **Interaction term / test** | **F (Num DF, Den DF)** | **p (joint)** | **OR_int (95% CI)** | **p (term)** |
| --- | --- | --- | --- | --- |
| **A. Modes of cannabis use × Age interaction** | | | | |
| **Joint test:** Modes of Cannabis use x Age | 1.12 (9, 42) | 0.3709 | - | - |
| Multi-mode × 18–25 | - | - | 1.36 (0.50, 3.72) | 0.5452 |
| Multi-mode × 26–34 | - | - | 0.93 (0.35, 2.49) | 0.8862 |
| Multi-mode × 35–49 | - | - | 1.61 (0.63, 4.12) | 0.3122 |
| Smoke-only × 18–25 | - | - | 0.89 (0.32, 2.49) | 0.8224 |
| Smoke-only × 26–34 | - | - | 0.73 (0.28, 1.90) | 0.5066 |
| Smoke-only × 35–49 | - | - | 1.50 (0.62, 3.65) | 0.3630 |
| Vape/dab-only × 18–25 | - | - | 2.55 (0.58, 11.16) | 0.2078 |
| Vape/dab-only × 26–34 | - | - | 1.46 (0.31, 6.79) | 0.6244 |
| Vape/dab-only × 35–49 | - | - | 3.56 (0.80, 15.83) | 0.0943 |
| **B. Modes of cannabis use × Sex interaction** | | | | |
| **Joint test:** Modes of cannabis use × Sex | 2.74 (3, 48) | 0.0534 | — | — |
| Multi-mode× Male | - | - | 0.61 (0.31, 1.21) | 0.1548 |
| Smoke-only × Male | - | - | 0.52 (0.25, 1.10) | 0.0877 |
| Vape/Dab-only × Male | - | - | 1.05 (0.40, 2.71) | 0.9255 |

**Notes:**

1. Joint tests are design-adjusted (Taylor series) F-tests from PROC SURVEYLOGISTIC.
2. OR_int: Odds ratios for the interaction.
